# Humidity Effect on Low-Temperature NH_3_ Sensing Behavior of In_2_O_3_/rGO Composites under UV Activation

**DOI:** 10.3390/s23031517

**Published:** 2023-01-30

**Authors:** Abulkosim Nasriddinov, Tatiana Shatalova, Sergey Maksimov, Xiaogan Li, Marina Rumyantseva

**Affiliations:** 1 Chemistry Department, Moscow State University, Moscow 119991, Russia; 2 Key Lab of Liaoning for Integrated Circuits Technology, School of Microelectronics, Dalian University of Technology, Dalian 116024, China

**Keywords:** metal oxide gas sensor, nanocrystalline indium oxide, reduced graphene oxide, UV activation, humidity effect, low temperature detection, NH_3_ sensor

## Abstract

The nature of the constituent components of composite materials can significantly affect the character of their interaction with the gas phase. In this work, nanocrystalline In_2_O_3_ was synthesized by the chemical precipitation method and was modified using reduced graphene oxide (rGO). The obtained composites were characterized by several analysis techniques—XRD, TEM, SEM, FTIR and Raman spectroscopy, XPS, TGA, and DRIFTS. The XPS and FTIR and Raman spectroscopy results suggested the formation of interfacial contact between In_2_O_3_ and rGO. The results of the gas sensor’s properties showed that additional UV illumination led to a decrease in resistance and an increase in sensor response at room temperature. However, the presence of humidity at room temperature led to the disappearance of the response for pure In_2_O_3_, while for the composites, an inversion of the sensor response toward ammonia was observed. The main reason may have been the formation of NH_4_NO_3_ intermediates with further hydrolysis and decomposition under light illumination with the formation of nitrite and nitrate species. The presence of these species was verified by in situ DRIFT spectroscopy. Their strong electron-accepting properties lead to an increase in resistance, which possibly affected the sensor signal’s inversion.

## 1. Introduction

Resistive-type gas sensors based on wide-gap semiconductor oxides are widely used in practical applications. A variety of commercial types are available on the market for the detection of toxic, polluting, and explosive gases [1,2]. However, the main effort of researchers is aimed at eliminating and minimizing the existing disadvantages, such as the thermal degradation of the sensitive layer, high power consumption, negative effects of humidity, and low selectivity [3,4,5,6,7,8]. The commonly used metal oxide semiconductors (MOSs), such as SnO_2_, In_2_O_3_, ZnO, WO_3_, TiO_2_, etc., show significant sensitivity toward different pollutant gases, but their high electrical resistance limits their gas-sensing performances at low operating temperatures. Many recent studies highlighted the use of perspective strategies to improve sensor performance: the use of modifiers depending on the nature of the analyte [9,10], applying filters [11], the use of photoactivation [12,13,14,15], and heterostructure creation [16,17,18].

In this regard, van der Waals two-dimensional (2D) materials, heterostructures, and devices, such as graphene and transition metal dichalcogenides, due to their unusual electronic and optical characteristics, can be quite effective [19,20,21,22]. The main advantages are their flexibility to change their electronic properties, in particular, the high electron mobility at room temperature for graphene, which allows the creation of p–n junctions, as well as the dependence of the band gap in such materials on the number of layers, which allows the control of the spectral characteristics of the resulting materials [23,24]. Previously, it was shown that the formation of a heterostructure between MOS and graphene can lead to efficient charge transfer, leading to an improvement in photocatalytic activity [25,26,27,28,29].

It should be noted that composite heterostructures of MOS with 2D materials, including graphene and its derivatives, have also shown promise in the field of gas sensors [30,31]. Hence, Quang et al. showed that tuning the Schottky barrier height and barrier width at the tiny area of contact between graphene and a SnO_2_ nanowire through the adsorption/desorption of gas molecules led to outstanding gas-sensing characteristics [32]. Shekhirev et al. studied CVD-grown graphene nanoribbon films that could reliably recognize VOCs from different chemical classes [33]. Abideen et al. [34] developed rGO nanosheet-loaded ZnO nanofibers with significantly higher responses toward different oxidizing and reducing gases than pure ZnO. This enhancement was proposed to be due to the creation of local p–n heterojunctions. Similarly, Tammanoon et al. [35] achieved a sensitive and selective NO_2_ sensor made from an electrolytically exfoliated graphene/flame-spray-made SnO_2_ composite operated at low temperatures. In combination with In_2_O_3_, which is highly chemically stable and has a large number of free charge carriers in the conduction band, surface oxygen vacancies, and active chemisorbed oxygen, such composites are able to effectively detect gases at room temperature [36,37,38,39]. On one hand, ammonia is widely used in various fields, including the agricultural, medical, and chemical industries. On the other hand, the production of large volumes leads to an increase in its concentration in the environment, which negatively affects human health. Thus, according to the NIOSH (National Institute for Occupational Safety and Health) for NH_3_, the TWA (time-weighted average concentration for up to a 10 h workday during a 40 h workweek) is 25 ppm and the ST (short-term exposure limit) is 35 ppm [40].

Nevertheless, it is also important to study the influence of light irradiation on the processes occurring at the solid–gas interface. Light radiation with the corresponding emission energy can lead to an increase in the concentration of charge carriers in a semiconductor matrix due to the generation of an electron–hole pair, to a change in the type and concentration of surface adsorption centers, and also to the formation of highly active radical particles that contribute to the oxidation of analyte molecules. All of the above can contribute to an increase in the sensitivity of sensors by accelerating ongoing processes. In particular, photogenerated holes can recombine with electrons localized on chemisorbed particles and lead to a decrease in the recovery time. In addition, for future practical applications, it is important to know the nature and character of the interaction of gas molecules with the material of the sensitive layer under different operating conditions, including high humidity values.

Herein, in this work, we obtained In_2_O_3_ and In_2_O_3_/rGO nanocomposites with varying rGO contents and conducted a systematic analysis of the chemical composition and interfacial interactions between components. The research explored the simultaneous influence of light irradiation and relative humidity, which can simulate the environmental conditions outside. The experimental results show that UV activation can enhance the sensing response toward NH_3_ at room temperature. The effect of humidity appears as an inversion of the sensor signal. A possible reason may be the photochemical conversion of surface NH_4_NO_3_ to nitrite and nitrate species. This study provides useful information for further understanding the influence of complex conditions on the sensing behavior of composite materials at room temperature.

## 2. Materials and Methods

### 2.1. Material Synthesis

#### 2.1.1. Synthesis of Nanocrystalline In_2_O_3_

The synthesis of the In_2_O_3_ nanocrystalline semiconductor oxide was performed via the chemical precipitation method. The synthesis procedure consisted of the precipitation of indium (III) hydroxide from an In(NO_3_)_3_ aqueous solution of its salt (5.00 g of In(NO_3_)_3_ × 4.5 H_2_O in 50 mL of deionized water). Precipitation was carried out by adding 13.3 M ammonia solution dropwise until pH ~ 6 was reached. The obtained dense gel was repeatedly washed several times and precipitated by centrifugation (8000× *g* rpm for 4 min) with 25 mM NH_4_NO_3_ solution and finally with deionized water to remove side products. The quality of purification from nitrate groups was checked using the Quantofix indicator (Macherey-Nagel, Düren, Germany). The resulting product was dried at T = 50 °C and then annealed at T = 300 °C for 24 h in air.

#### 2.1.2. Synthesis of Composite Materials

Commercial rGO from “Lition Company” (Dubna, Russia) was used to obtain the composite materials. rGO was synthesized by a modified Hummer’s method, then dispersed and stored in ethylene glycol to maintain the degree of reduction. A pre-weighed amount of the rGO suspension was added to the In_2_O_3_ powder and further diluted with 1 mL of citric acid (6.5 mM). The resulting mixture was stored for 20 min in an ultrasonic bath at a temperature of 50 °C to obtain a uniform distribution for the constituent components. The powder was dried at T = 50 °C and then annealed at T = 150 °C in air for 10 h. As a result, In_2_O_3_/rGO composites with a rGO content of 0.5 and 1.0 wt.% were obtained.

### 2.2. Materials Characterization

The phase composition of the as-prepared samples was characterized by X-ray powder diffraction (XRD) using a DRON-4 X-ray diffractometer (Burevestnik, Moscow, Russia) with Cu Ka radiation (λ = 1.5418 Å) and Raman spectroscopy using an i-Raman Plus spectrometer (BW Tek, Plainsboro, NJ, USA) equipped with a BAC 151C microscope and a laser with an excitation wavelength of 532 nm. The morphology and composition of the samples were analyzed by scanning electron microscopy (SEM) using a Carl Zeiss SUPRA 40 FE-SEM instrument (Carl Zeiss AG, Jena, Germany) with an Inlens SE detector. Transmission electron microscopy (TEM) studies were performed using the high-resolution electronic transmission microscope JEOL JEM-2100F at 200 kV.

Thermogravimetric analysis with mass spectral analysis of gaseous products (TG-MS) was carried out using a NETZSCH STA 449 instrument combined with a QMS-409 mass spectrometer (Netzsch-Gerätebau GmbH, Selb, Germany). The samples were heated in airflow (30 mL/min) at a rate of 10 °C/min in the temperature range of 25–700 °C. 

Infrared spectra were recorded on a Spectrum One Fourier-transform infrared spectrometer (Perkin Elmer Inc., Waltham, MA, USA) in the transmission mode and the wavenumber range of 400–4000 cm^−1^ with a step of 1 cm^−1^. Sample preparation consisted of pressing the samples (about 5 mg) with KBr (50 mg) to obtain tablets (6 mm diameter and 0.5 mm thickness). Diffuse reflectance infrared Fourier-transform spectroscopy (DRIFTS) was also performed on a Spectrum One Fourier-transform infrared spectrometer (Perkin Elmer Inc.) with the DiffusIR annex and flow chamber HC900 (Pike Technologies, Fitchburg, WI, USA) in the range of 4000–1000 cm^−1^ with a resolution of 4 cm^−1^ and accumulation of 30 scans. The samples (35 mg) were placed in ceramic crucibles (5.0 mm diameter, 3.5 mm depth) and placed into the heating chamber. The samples were preheated to 150 °C for 1 h to remove weakly adsorbed species and then cooled down to room temperature. The spectra were recorded at room temperature under a controlled flow rate of 100 mL/min in a gas mixture containing 100 ppm NH_3_ in dry air or with a relative humidity (RH) of 70%.

X-ray photoelectron spectroscopy (XPS) studies were carried out on an OMICRON ESCA+ spectrometer (Scienta Omicron, Uppsala, Sweden) with an aluminum anode equipped with an AlKα XM1000 monochromatic X-ray source (radiation energy 1486.6 eV and power 252 W). To eliminate the local charge on the analyzed surface, a CN-10 charge neutralizer with an emission current of 6 μA and beam energy of 1 eV was used. The analyzer transmission energy was 20 eV. The spectrometer was calibrated toward the Au4f 7/2 line at 84.1 eV. The pressure in the analyzer chamber did not exceed 10^−9^ mbar. All spectra were accumulated at least three times. The fluctuation in the position of the peaks did not exceed ±0.1 eV.

Specially designed micro-hotplates were used for resistance measurements. The micro-hotplates consisted of a dielectric substrate (0.9 mm × 0.9 mm × 0.15 mm) made from Al_2_O_3_. There were platinum electrodes for heating on the back side of the substrates, and the electrodes on the top side were intended to measure the resistance. The electrodes and heater were made using Pt-based paste by the screen-printing method. The synthesized samples in the form of powders were pre-dispersed with ethanol and deposited as a thick film on the surface of the dielectric substrate to cover the electrodes. The films were sintered at T = 150 °C in air for 5 h.

Gas sensor measurements were performed using laboratory-made equipment with a flow cell. DC measurements were carried out in the temperature range of 150–25 °C with a step of 25 °C. During the tests, the concentration of the analyte gas was controlled by mass flow controllers by mixing a certified gas mixture with purified air. Gas was supplied through the cell with a flow rate of 100 mL/min. The measurements were carried out with a cyclic change in the atmosphere’s composition: air with analyte for 15 min and pure air for 30 min. Preliminarily, for 100 min, the chamber with sensors was purged with purified air at a temperature of 150 °C. The sensor signal was calculated as:

$$S\;=\;\frac{R\left(air\right)-R\left(gas\right)}{R\left(gas\right)}\times 100\%,$$

where *R*(*air*) is the sensor’s resistance in air and *R*(*gas*) is the sensor’s resistance under a gas-containing atmosphere (NH_3_).

The relative humidity in the DRIFTS and sensor measurements was controlled by regulating the ratio of dry and wet stream flows and was registered by a hygrometer IVTM-7 (Practic-NC, Zelenograd, Russia). A UV light-emitting diode (LED, λ_max_ = 365 nm, P = 3.5 mW/cm^2^) was used for irradiation.

## 3. Results and Discussion

### 3.1. Characteristics of Composite Materials

**Figure 1:**
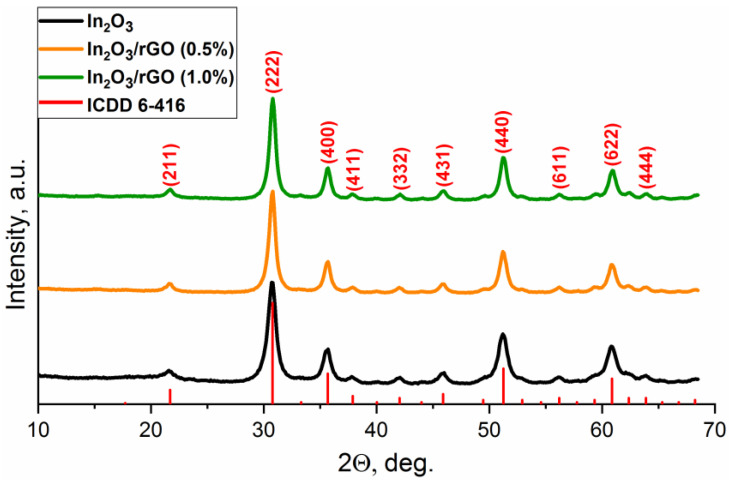
XRD patterns of the In_2_O_3_ and In_2_O_3_/rGO composites.

The phase composition of the obtained samples was characterized using ICDD PDF-2 for In_2_O_3_ (6-416) (Figure 1). The X-ray diffraction patterns corresponded to the In_2_O_3_ phase with the cubic bixbyite structure, which indicated that the synthesized sample was single-phase. The X-ray patterns of the composite materials did not have significant differences, suggesting no influences on the structural characteristics after modification. The particle sizes of indium oxide in the pure sample and composites, estimated by the Scherrer formula from the most intense peaks, were approximately equal and corresponded to 7–8 nm.

**Figure 2:**
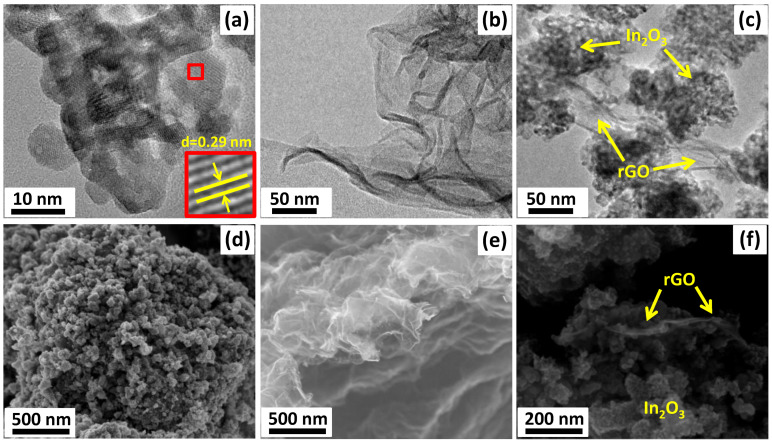
TEM images (**a**–**c**) and SEM images (**d**–**f**) of the In_2_O_3_ (**a**,**d**), rGO (**b**,**e**), and In_2_O_3_/rGO composite (**c**,**f**).

According to the TEM images (Figure 2a), indium oxide nanoparticles had a shape close to spherical with a size in the range of 7–10 nm, which was in good agreement with the values obtained from XRD data. In addition, crystalline particles with interplanar spacings d = 0.29 nm corresponding to the In_2_O_3_ (222) plane were found.

The rGO nanosheets (Figure 2b,e) had a typical 2D morphology and visually appeared to be crumpled due to tightly attached layers. According to the SEM, images the nanocrystalline In_2_O_3_ had a three-dimensional and porous structure with sintered grains (Figure 2d). The obtained composites consisted of agglomerated In_2_O_3_ nanoparticles attached by two-dimensional graphene flakes in the form of bridges (Figure 2c,f).

**Figure 3:**
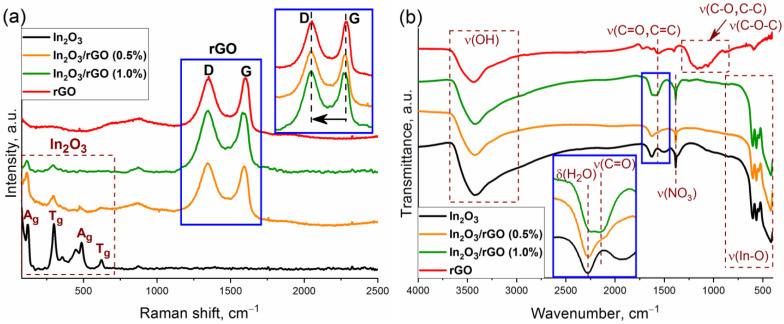
Raman spectra (**a**) and FTIR spectra (**b**) of the In_2_O_3_, rGO, and In_2_O_3_/rGO composites.

The structure of the semiconductor oxide and composite materials was also studied by Raman spectroscopy. Figure 3a shows the Raman spectra of nanocrystalline In_2_O_3_, rGO, and the composite materials in the frequency range of 90–2500 cm^−1^. Characteristic Raman modes corresponding to the body-centered cubic lattice of In_2_O_3_ were observed at 122.5, 298.5, 357.2, 487.6, and 621.5 cm^−1^. The vibration of the In-O bond of the [InO_6_] structural units was observed at 122.5 cm^−1^. The vibrational mode at 298.5 cm^−1^ was associated with a bending vibration of the [InO_6_] octahedra, while the modes at 487.6 and 621.5 cm^−1^ were attributed to the stretching vibrations of the [InO_6_] octahedra. The band at 357.2 cm^−1^ corresponded to the stretching vibrations of the In-O-In bonds [41,42,43]. The broad band at 447.3 cm^−1^ corresponded to surface structural defects due to the small particle size of nanocrystalline In_2_O_3_ [44].

Reduced graphene oxide has two characteristic vibrational modes at 1346.3 and 1599.8 cm^−1^, which are designated as D and G modes, respectively. The G-mode (E_2g_ symmetry) is due to the stretching vibrations of carbon atoms (C-C) in the plane. This peak can be observed in the Raman spectra for all carbon structures containing sp^2^ hybridized bonds, while the D-mode (A_1g_ symmetry) becomes active in the presence of any defect in the ideal structure. Therefore, it can be seen as a band caused by disturbances and it can be described as a “breathing oscillation” of hexagonal aromatic carbon rings [45]. For the In_2_O_3_/rGO (0.5%) and In_2_O_3_/rGO (1.0%) composite materials, the intensity of the A_g_ and T_g_ vibrational modes corresponding to indium oxide decreased with increasing rGO content. This may have been due to the fact that rGO, which had a black color, absorbed green laser radiation to a greater extent; therefore, scattering in it will occur with greater probability. The presence of D and G bands indicated the preservation of the rGO structure after synthesis and thermal treatment. Moreover, a red shift in the position of the G-band occurred with increasing rGO content in composites from 1599.8 cm^−1^ (for rGO) to 1592.6 cm^−1^ (for In_2_O_3_/rGO (0.5%)) and to 1582.8 cm^−1^ (for In_2_O_3_/rGO (1%)). This gradual shift may indicate a charge transfer as a result of chemical bond formation with the surface of In_2_O_3_ [46]. 

The level of disorder in graphene oxide can be qualitatively estimated from the ratio of the intensities of the D and G bands (I_D_/I_G_). From pure rGO (I_D_/I_G_ = 0.98) to composites (I_D_/I_G_ = 1.02 and 1.04), an increase in this ratio was observed, which may have indicated an increase in the surface oxygen-containing functional groups that formed upon bonding with a semiconductor oxide [47,48]. The increase in the I_D_/I_G_ ratio may have also indicated a decrease in the number of graphene layers [27].

Figure 3b shows the results of the study of samples by FTIR spectroscopy. The peaks of adsorbed water (1628 cm^−1^) and hydroxyl groups (3000–3670 cm^−1^) can be observed in the spectra. In the low-frequency region, In_2_O_3_ and composite materials exhibited characteristic peaks corresponding to the vibrations of the In-O bonds in the crystal lattice [49]. The presence of vibration bands corresponding to nitrate ions (1385 cm^−1^) may have been due to residual impurities from the precursor that could not be washed during the synthesis procedure.

The spectrum of rGO is characterized by the presence of vibration bands with weak intensities, which most likely belong to different surface functional groups: hydroxyl (C–OH), epoxy (C–O–C), carbonyl (C=O), or carboxyl (COOH). Thus, a wide band in the region of 910–1320 cm^−1^ corresponds to the superposition of stretching vibrations of the (C-O), (C-C), and (C-O-C) bonds. The bands at 580, 1400, 1565, and 1720 cm^−1^ were associated with vibrations of the (C-C), (-COOH), (C=C), and (C=O) bonds, respectively [49,50,51].

At the same time, the FTIR spectra of the composites were characterized by the appearance of a new peak at 1576 cm^−1^ (C=O or C=C). The intensity of this peak increased with an increase in the rGO content. The obtained results indicated the efficient immobilization of rGO with the semiconductor oxide. Such contact can provide an electronic interaction between In_2_O_3_ and rGO, which can lead to better charge separation.

**Figure 4:**
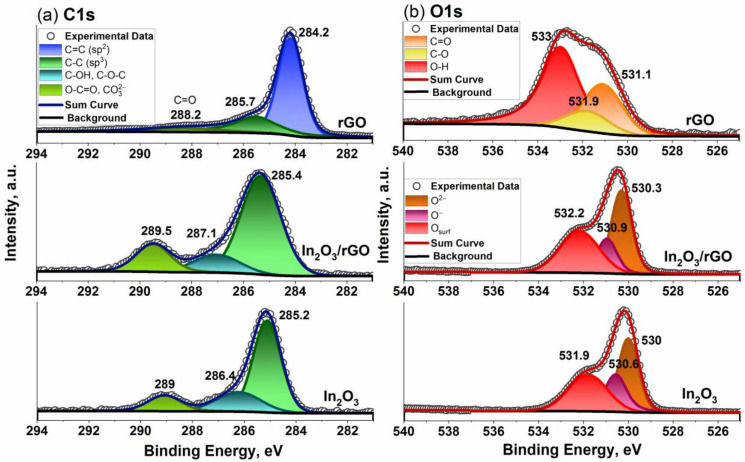
C 1s (**a**) and O 1s (**b**) X-ray photoelectron spectra of the In_2_O_3_ and In_2_O_3_/rGO (1%) composite.

XP spectra of the samples are shown in Figure 4. For the rGO sample, both the C 1s and O 1s spectra differed from those of pure In_2_O_3_ and the In_2_O_3_/rGO (1%) composite. The deconvolution of the C 1s spectrum showed that it consisted of sp^2^ bonding C=C, sp^3^ bonding C-C, and C=O (or O-C=O) bonds at 284.2, 285.7, and 288.2 eV, respectively [52]. The O 1s spectrum consisted of the following three components: a C=O (carbonyl and carboxyl) bond at 531.1 eV, a C-O (epoxy) bond at 531.9 eV, and an O-H (hydroxyl) bond at 533 eV [53]. The C1s spectrum of the pure In_2_O_3_ sample consisted of several components of adventitious carbon. Thus, an intense peak at 285.2 eV corresponded to sp^3^ bonding C-C, the broad peak at 286.4 eV was due to C-OH and C-O-C bonds, while the peak at 289 eV corresponded to the O-C=O bond or carbonate contamination ${\mathrm{CO}}_{3}^{2-}$ [54]. For the In_2_O_3_/rGO (1%) composite sample, a shift and an increase in the quantitative ratio of these components could be observed, which may have indicated an interaction between In_2_O_3_ and rGO. An additional shift of 0.3 eV for the In 3d spectrum toward higher binding energies also indicated charge redistribution and transfer at the interface.

The O1s spectra of pure In_2_O_3_ could be fitted using three peaks with maxima at binding energies of 530, 530.6, and 531.9 eV. The lowest binding energy region was associated with lattice oxygen (O^2−^), while the middle region could be assigned to oxygen ions with lower electron density (O^−^) in the subsurface. The coordination number of the oxygen ions in these sites was lower and it could indicate the defective structure of the metal oxide [55]. The appearance of the peaks in the higher-binding-energy region was due to chemically adsorbed oxygen-containing species on the sample’s surface [56,57]. These peaks shifted by 0.3 eV to the higher binding energy for the In_2_O_3_/rGO (1%) composite. Moreover, the ratio of their content (O^2−^:O^−^:O_surf_) changed between pure In_2_O_3_ (40:24:36) and the composite (41:20:39), which indicated the compensation of oxygen deficiencies leading to an increase in surface species, including chemisorbed oxygen ions.

**Figure 5:**
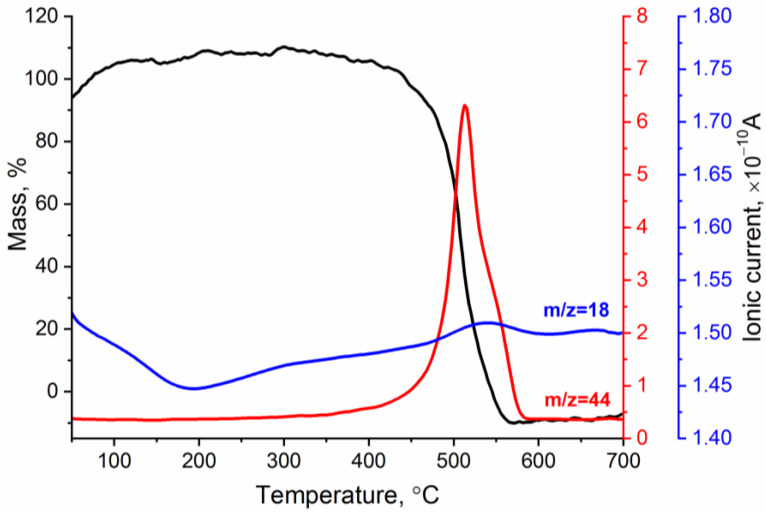
TG curve and temperature dependencies of ionic currents corresponding to H_2_O (*m*/*z* = 18) and CO_2_ (*m*/*z* = 44) during rGO fragmentation and oxidation.

Figure 5 shows the TG curve and temperature dependencies of the ionic currents corresponding to gaseous products released from the reduced graphene oxide. The analysis showed that, in the temperature range of 50–200 °C, there was a slight increase in the mass of the sample, which was accompanied by a decrease in the ion currents for *m*/*z* = 18 (H_2_O). Most likely, rGO was partially oxidized at this stage. At T = 450 °C, the destruction of the rGO skeleton began: C–C bonds were broken, resulting in the formation of CO_2_ (*m*/*z* = 44). It should be noted that, in this temperature range, the mass loss was almost 100%.

### 3.2. Gas Sensor Measurements

**Figure 6:**
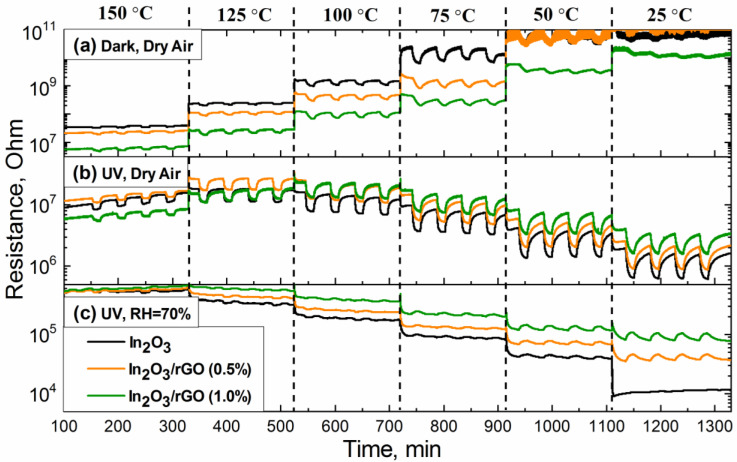
Dynamic changes in sensors’ resistance with periodic changes in the gas phase composition (20 ppm NH_3_–purified air) in the temperature range of 150–25 °C under various experimental conditions: (**a**) under dark conditions with dry air; (**b**) under UV illumination with dry air; and (**c**) under UV illumination with a relative humidity of 70% (RH = 70%).

Figure 6 shows the change in the resistance of the obtained sensors depending on the gas phase composition under different conditions. It is noteworthy that the sensors did not exhibit baseline drift across the entire measurement range, even under a humid atmosphere. It can be seen that, under an atmosphere of dry air (Figure 6a,b), all samples behaved like n-type semiconductors: when 20 ppm of ammonia (reducing gas) was added, the resistance decreased due to reaction (1), and under an atmosphere of purified air, the resistance increased again as reaction (2) could proceed.

(1) $$2NH_{3\left(gas\right)}+\frac{3}{\beta }O_{\beta \left(ads\right)}^{\alpha -}\to N_{2\left(gas\right)}+3H_{2}O_{\left(gas\right)}+\frac{3\alpha }{\beta }e^{-},$$

(2) $$\frac{\beta }{2}O_{2\left(gas\right)}+\alpha e^{-}\to O_{\beta \left(ads\right)}^{\alpha -}$$

where $NH_{3\left(gas\right)}$ represents the ammonia molecules in the gas phase and $O_{\beta \left(ads\right)}^{-\alpha }$ represents chemisorbed oxygen species (*α* = 1 and 2 for once- and twice-charged particles, respectively; *β* = 1 and 2 for atomic and molecular forms, respectively).

A high electrical conductivity of the rGO compared with MOS led to a decrease in the baseline resistance for composites under dark conditions (Figure 6a) [22]. As the resistance of the semiconductor materials increased with decreasing temperature, at low operating temperatures (25 and 50 °C), the resistance value reached above the detection limit of the device (R > 10^10^ Ohm), so the data were noisy and illegible, which led to measurement and calculation difficulties.

**Figure 7:**
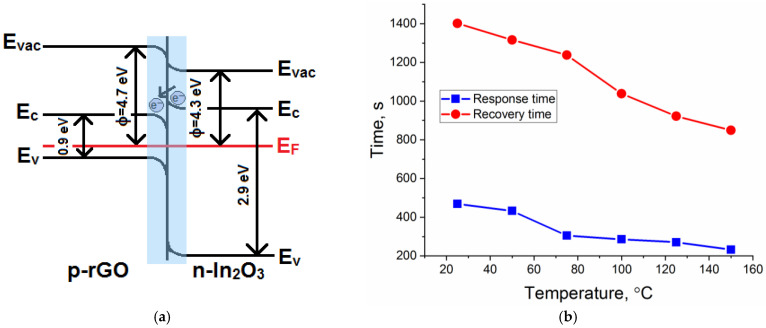
A schematic illustration of the p–n heterojunction formation in the rGO–In_2_O_3_ interface (**a**); response and recovery times of the In_2_O_3_/rGO (1%) composite under UV illumination at different operating temperatures (**b**).

**Figure 8:**
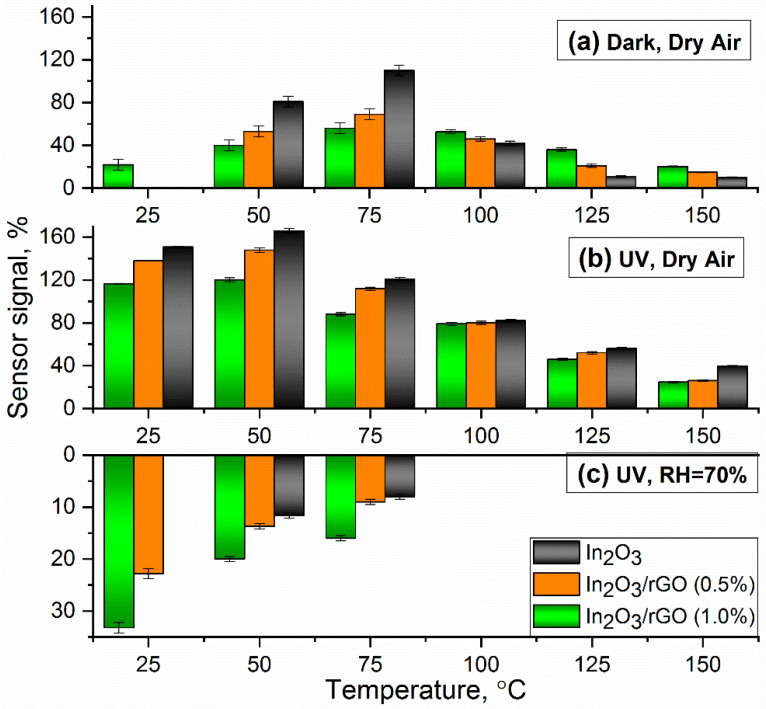
Temperature dependence of the sensor signal of the materials upon the detection of 20 ppm NH_3_ under various experimental conditions: (**a**) under dark conditions with dry air; (**b**) under UV illumination with dry air; and (**c**) under UV illumination with a relative humidity of 70% (RH = 70%).

As illustrated in Figure 7a, the addition of p-type rGO to the n-type In_2_O_3_ semiconductor matrix formed a p-to-n transition at the interface of the obtained composite. Electrons would be transferred from In_2_O_3_ with a lower work function (4.3 eV) to rGO with a higher work function (4.7 eV), resulting in depletion layer formation until the equilibrium of the Fermi level was reached [58,59,60,61]. As a result, a potential barrier would be created at the heterojunction. The formation of p–n heterojunction led to an increase in the resistance value of the composites, which could be observed based on the dependences of the resistance over time (Figure 6b,c). In addition, it could significantly reduce the rate of recombination of electron–hole pairs and promote their separation and migration to the semiconductor surface under the action of internal electric fields. Figure 7b represents the response and recovery time for the In_2_O_3_/rGO (1%) composite to 20 ppm NH_3_ under UV illumination. The results show that increasing the operating temperature led to a decrease in both the response and recovery times. At the same time, comparing with Figure 8, one can notice that the slower the reaction preceded, the higher the sensor signal. This was achieved in the low temperature range, while at higher temperatures, partial desorption of oxygen from the surface may have occurred.

In the case of UV illumination (Figure 6b), a decrease in resistance of all sensors was observed, thereby achieving a reproducible response to NH_3_, even at room temperature. When In_2_O_3_ semiconductor particles were exposed to UV irradiation, photogenerated electrons and holes were formed (3). An increase in the concentration of photoelectrons in the conduction band led to a decrease in the base resistance of the In_2_O_3_ sample, particularly compared with composites. In the latter case, rGO flakes could cover the surface of the semiconductor, thereby preventing the interaction of UV radiation with the semiconductor’s solid surface. An increase in the concentration of charge carriers in the conduction band could enhance the adsorption of oxygen from the atmosphere (reaction 2), which, in turn, could stimulate reaction (1). Acting as an electron acceptor, rGO could prevent the rapid recombination of electrons and holes [62].

(3) $$In_{2}O_{3}\overset{h\nu }{\to }e^{-}+h^{+}$$

Nevertheless, in the first two cases, the unmodified In_2_O_3_ exhibited a higher sensor signal compared with the composite materials (Figure 8a,b). However, it is worth noting that UV illumination significantly improved the response to NH_3_, especially in the low-temperature region.

An interesting case is the third one (Figure 6c and Figure 8c), when the measurements were carried out under an atmosphere with RH = 70%. First, it can be noticed that the baseline resistance of the sensors decreased by more than one order of magnitude compared with the measurements under a dry atmosphere. It is mentioned in the literature that this phenomenon may have been associated with an increase in the electron concentration in the conduction band due to the following reactions [63]:

*H*_2_*O_(gas)_* + *In_(lat)_* + *O_(lat)_* = *[In_(lat)_* − *OH]* + *[O_(lat)_H]*∙∙ + *e*^−^ (4)

*H*_2_*O_(gas)_* + *2In_(lat)_* + *O_(lat)_* = *2[In_(lat)_ − OH]* + *V_O_*∙∙ + *2e*^−^ (5)

*2H*_2_*O_(gas)_* + *4In_(lat)_* + *O*_2_^−^*_(ads)_* = *4[In_(lat)_ − OH]* + *e*^−^ (6)

In the temperature range of 100–150 °C, the resistance of the sensors fluctuated at the noise level and there were no significant changes, indicating the negative effect of water vapor on the detection of ammonia and the lack of charge transfer occurring. In the low temperature range (25–75 °C), the effect of inversion of the sensor signal was observed, and the lower the measurement temperature, the better this effect revealed itself. This situation implies that, under an atmosphere containing ammonia, the resistance increased, and under an atmosphere of purified air, it decreased.

**Figure 9:**
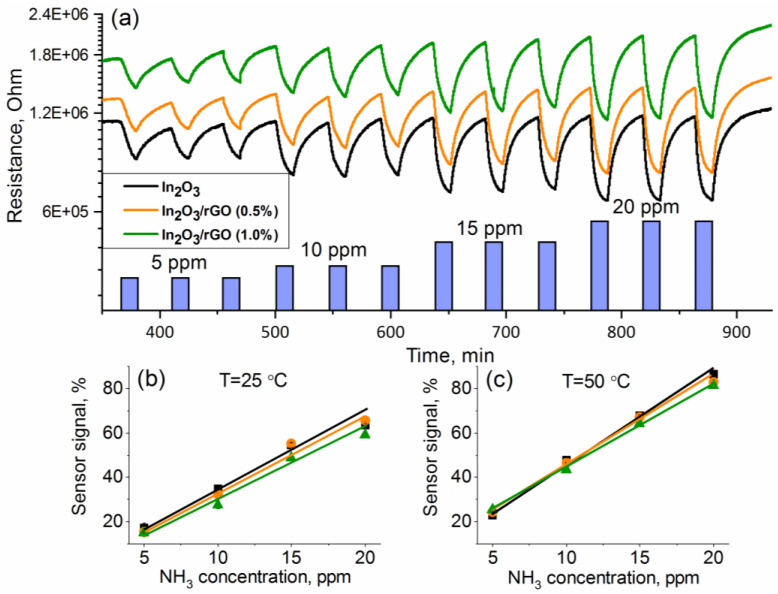
Change in the samples’ resistances depending on the NH_3_ concentration (5–10–15–20 ppm) (**a**); calibration curves at T = 25 °C (**b**) and T = 50 °C (**c**).

The minimum detectable NH_3_ concentration was calculated by plotting calibration curves, which had a good linear relationship with the ammonia concentration (Figure 9). The minimum measurable sensor response was estimated using the ratio of *R(av)/(R(av) − 3σ*), where *R(av)* is the average resistance in pure air and *σ* is the standard deviation of resistance in pure air. The noise level of the sensors was calculated as the changes in the relative response of the sensor over the baseline or the root-mean-square deviation (RMS_noise_). Sensitivity was determined as ΔR/Δc. The obtained results are presented in Table 1. It can be observed that the In_2_O_3_/rGO (1%) composite demonstrated the lowest value of the minimum detectable NH_3_ concentration, RMS_noise_, and sensitivity at T = 50 °C. However, it should be noted that the obtained values were quite close.

### 3.3. In Situ DRIFTS Analysis

**Figure 10:**
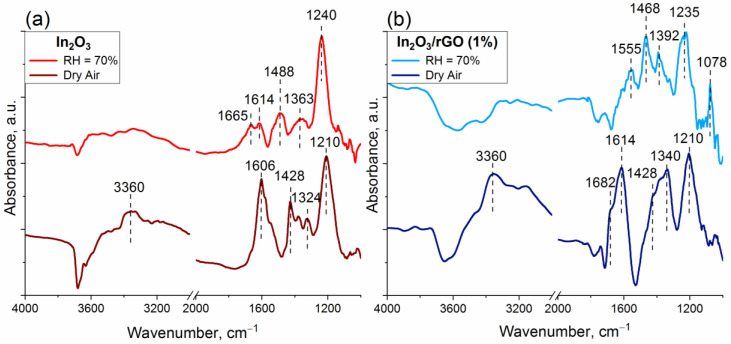
In situ DRIFT spectra of the In_2_O_3_ (**a**) and In_2_O_3_/rGO (1%) (**b**) samples after 100 ppm NH_3_ adsorption for 50 min at room temperature in dry air and humid air (RH = 70%).

In order to analyze in more detail the effect of humidity on the nature of the change in resistance, a study entailing DRIFT spectroscopy was carried out. The spectra were recorded at room temperature in dry air and humid air (RH = 70%). The samples were preliminarily kept under a flow of purified air at T = 150 °C for 40 min. The results are shown in Figure 10.

After 5 min of NH_3_ exposure, narrow peaks in the range of 1210–1240 cm^−1^ were immediately detected. These bands were assigned to NH_3_^+^ species adsorbed on Lewis acid sites. An additional confirmation may be offered by the appearance of N-H stretching vibrations at the wavenumber of 3360 cm^−1^ related to NH_3_^+^ species under an atmosphere of dry air. The appearance of bands in the range of 1428–1488 cm^−1^ was associated with NH_4_^+^ species as a result of ammonia adsorption on Brønsted acid sites, including terminal In-OH groups. Such adsorption was accompanied by a decrease in the intensity of the bands of OH groups [49,64].

Intense peaks corresponding to molecularly adsorbed NH_3_ on Lewis acid sites in the range of 1606–1682 cm^−1^ appeared only under the dry atmosphere, while under humid conditions, they disappeared or decreased in intensity [65]. This may indicate, by implication, the predominant coverage of the surface and, accordingly, the occupation of active sites by water molecules. However, the IR band corresponding to NH_3_^+^ did not change in intensity both under dry and humid air, which could be due to stronger interaction with Lewis acid sites compared with hydroxyl groups.

It can be noticed from Figure 3b that residual nitrate groups remained in the composition of the samples after synthesis due to the precursor. In the presence of ammonia, they could react with the formation of a surface intermediate, likely NH_4_^+^–NO_3_^−^ species. This was confirmed by the appearance of absorption bands in the region of 1324–1392 cm^−1^. Previously, the same surface species with similar vibration frequencies were also detected on different catalysts: during the reaction between NO_2_ and a NH_3_-pre-adsorbed Cu-exchanged SAPO-34 catalyst [65], a V_2_O_5_–WO_3_/TiO_2_ catalyst after exposure to NH_3_ and NO_2_ [66], and an Fe-zeolite-based catalyst after exposure to NO_2_ over a NH_3_-pre-adsorbed sample [67]. The main characteristic infrared vibrational frequencies, which were found according to the results of DRIFTS analysis, are shown in Table 2 [49,64,68,69].

From Figure 10b, it is clear that, after NH_3_ was introduced to the chamber with 70% background humidity, new bands at 1078 and 1555 cm^−1^ appeared. These bands could be attributed to monodentate nitrite and chelating bidentate nitrate species. However, the exact identification of these bands is an ambiguous task due to the overlapping of the absorption regions of various structural fragments, including nitrates and nitrites (both monodentate and bidentate species) [68,69].

It can be observed from Figure 6c that the effect of sensor signal inversion was observed, even for pure In_2_O_3_ at T = 50 and 75 °C under a humid atmosphere, while at T = 25 °C, it was observed exclusively for composites. Hence, it can be assumed that the main factor affecting the signal inversion was the indium oxide matrix. From the DRIFTS results, it was found that the interaction with ammonia resulted in the formation of NH_4_NO_3_ species. It could be assumed that further interaction of this fragment with water molecules in a sufficiently high humid atmosphere (RH = 70%) led to the formation of nitric acid. The reaction pathway might have involved NH_4_NO_3_ hydrolysis on the surface of particles. The formed intermediate nitric acid may have been decomposed under light illumination to produce NO_2_ [70,71,72,73,74]. In the case of the In_2_O_3_/rGO composites, rGO flakes could serve as an additional path for charge transfer due to heterocontact and side reactions on the surface.

Recently, Ma et al. comprehensively investigated the photolysis of various nitrates on different mineral oxides [75]. It was found that NH_4_NO_3_ had the highest rate of NO_2_ production, even at room temperature, compared with the other studied nitrates. Moreover, this rate was higher under a humid atmosphere compared with dry air. The authors used UV irradiation with 365 nm wavelength (as in this work) and assumed that photoinduced electrons and holes (reaction (3)) could promote the photolysis of NH_4_NO_3_ on the metal oxide’s surface (reactions (7) and (8)). The photochemical reaction between the water and photogenerated hole could enhance the surface acidity and facilitate NO_2_ production.

(7) $$NO_{3}^{-}+h^{+}\to NO_{3}$$

(8) $$2NO_{3}\overset{h\nu }{\to }2NO_{2}\;+O_{2}$$

Nitrogen dioxide, being a strong electron acceptor, can attract electrons from the conduction band, thereby leading to an increase in the resistance of the sensors and consequently inverting the signal (reaction (9)). The electron affinity of NO_2_ (2.27 eV, [76]) is greater than that of O_2_ (0.44 eV, [77]). Therefore, in the subsequent competing process between NO_2_ and O_2_ (according to the products of reaction (5)), nitrogen dioxide will predominate. It is also worth noting that the signal inversion is reproducible (Figure 6c), which may indicate the regeneration of NH_4_NO_3_. The formation of nitrate species can proceed via reaction (10) and, according to the DRIFTS results, further interaction with ammonia can lead to NH_4_NO_3_ formation again.

(9) $$NO_{2}+e^{-}\to NO_{2}^{-}$$

(10) $$2NO_{2}\;+O_{2}+e^{-}\to 2NO_{3}^{-}$$

## 4. Conclusions

Composite materials based on nanocrystalline In_2_O_3_ and rGO were synthesized and investigated. The obtained Raman and FTIR spectroscopy results indicate the efficient immobilization of rGO with a semiconductor oxide matrix. The influence of UV activation and humidity on the gas-sensing behavior of In_2_O_3_/rGO composites was also studied.

When ammonia interacted with composite materials, the main adsorption sites were provided by the porous surface of In_2_O_3_. Additional surface modification with rGO, which consisted of flakes with large lateral sizes, could limit the access to and interaction with the analyte gas molecules, resulting in a reduced sensor signal in dry air. However, UV illumination led to the generation of electron–hole pairs in the In_2_O_3_ structure, as this energy was comparable to its band gap. An increase in the electron concentration in the conduction band promoted greater oxygen adsorption and, accordingly, more efficient interaction with ammonia. At the same time, under an atmosphere with high relative humidity, the predominant active centers, including chemisorbed oxygen, were occupied or replaced by adsorbed water. On one hand, this led to an increase in conductivity, and on the other hand, the decrease in the concentration of chemisorbed oxygen limited the oxidation of ammonia and further charge transfer. As a result, the sensor signal for pure In_2_O_3_ was noticeably reduced.

Based on the in situ DRIFTS analysis, it is proposed that residual nitrate groups can react with ammonia, resulting in the formation of surface intermediates, likely NH_4_NO_3_ species. The combined influence of humidity and UV illumination could lead to the hydrolysis of NH_4_NO_3_ on the In_2_O_3_ surface, followed by photolysis, or immediately undergo a photochemical reaction. As a result, nitrite and nitrate species were formed. Due to their electron-accepting nature, they led to a decrease in conductivity, resulting in an inversion of the sensor signal when detecting ammonia at low temperatures. The appearance of these groups was proven by DRIFT spectroscopy. The effect of signal inversion was most clearly expressed for a composite with a rGO content of 1% at room temperature in RH = 70%. In this case, rGO flakes could serve as an additional path for charge transfer due to heterocontact and side reactions on the surface.

## Figures and Tables

**Table 1:** Minimum detectable NH_3_ concentration c_min_, RMS_noise_, and sensitivity of the sensors measured at 25 °C and 50 °C under dry air conditions and UV illumination.

Sample	T = 25 °C			T = 50 °C		
	C_min_, ppm	RMS_noise_, × 10^−3^	Sensitivity, ppb^−1^	C_min_, ppm	RMS_noise_, × 10^−3^	Sensitivity, ppb^−1^
In_2_O_3_	1.71	18.02	31.5	1.1	14.12	38.5
In_2_O_3_/rGO (0.5%)	1.78	20.65	34.7	1.04	13.5	38.8
In_2_O_3_/rGO (1%)	1.88	21.12	33.6	1.0	12.53	37.4

**Table 2:** Assignments of IR absorption bands (cm^−1^) that appeared in the DRIFT spectra on the surface of In_2_O_3_ and In_2_O_3_/rGO (1%) samples under different conditions.

Functional Groups	In_2_O_3_		In_2_O_3_/rGO (1%)	
	Dry Air	RH = 70%	Dry Air	RH = 70%
NO_2_^−^, monodentate nitrite	-	-	-	1078
NH_3_^+^ on Lewis acid site	1210	1240	1210	1235
ν(NO_3_) in NH_4_NO_3_ species	1324, 1378	1363	1340, 1380	1392
NH_4_^+^ on Brønsted acid site	1428	1488	1428	1468
NO_3_^−^, chelating bidentate nitrate	-	-	-	1555
NH_3_, molecularly adsorbed on Lewis acid sites	1606	1614, 1665	1614, 1682	-
ν(N-H) in NH_3_	3360	-	3360	-
ν(OH)	3530–3715	3600–3715	3450–3740	3320–3820

## Data Availability

The data that support the findings of this study are available from the corresponding author upon reasonable request.
